# Microbes set the (woodrat) menu: Host genetics control diet-specific gut microbes

**DOI:** 10.1073/pnas.2120125118

**Published:** 2021-12-23

**Authors:** Taichi A. Suzuki, Ruth E. Ley

**Affiliations:** ^a^Department of Microbiome Science, Max Planck Institute for Biology, Tübingen 72076, Germany

The mammalian gut is a great place for microbes: a constant warm temperature, predictable moisture and pH, and—if life is good for the host—a steady supply of food. In return for such a rich habitat, microbes perform key services such as fiber digestion. This is critical for the vast majority of mammals, whose genomes do not encode the enzymes required to degrade the plant polysaccharides. Indeed, the evolution of mammals is deeply entwined with the history of the gut symbionts that enable the consumption of all types of plants. One intriguing aspect of microbial diversity across mammals is that related species often harbor similar gut microbial communities, a pattern termed phylosymbiosis (1). The root causes of phylosymbiosis, and the implications of this process for host–microbial interactions and coevolution, are largely unexplored.

Although phylosymbiosis readily emerges from microbiome surveys of mammals (2), it is not readily explained, as many of the factors that can drive such a pattern are confounded. Is phylosymbiosis the result of shared diet, shared habitat, or the genetic relatedness of the hosts? The gut is an open system, technically outside the body, and the microbiota are acquired from the environment after birth, so gut microbes can spread between host species. Closely related host species often have similar diets (e.g., try to find a carnivorous ungulate), and many live in the same environments; therefore, similar microbiomes may track with similar diets or shared environments, rather than with the genetic relatedness of the host species. In PNAS, Weinstein et al. (3) make a significant step toward untangling how host phylogeny, diet, and shared environment explain phylosymbiosis.

**Fig. 1.**
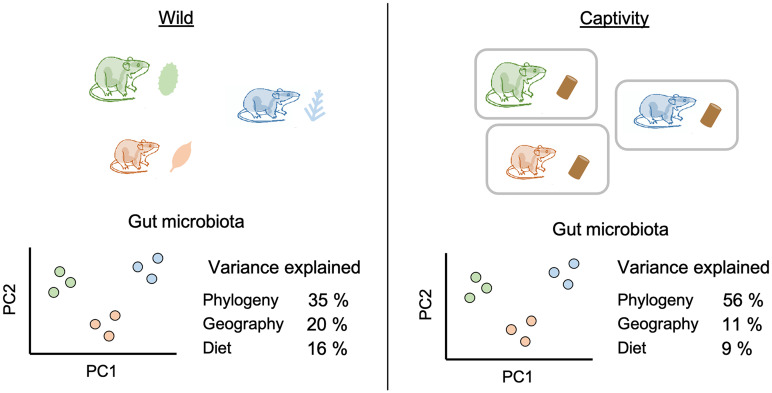
A graphical summary of the study design (3). Microbiomes of seven species of woodrats (*Neotoma* spp.) that feed on different compositions of plant species were studied in the field. The animals were then brought into captivity and fed a standardized diet. Host phylogeny explained the greatest proportion of variation of the woodrat microbiotas in the wild and in captivity, relative to geographical origin and diet. Principal coordinate plots of the microbiota are graphical representations of the results and do not reflect original data. The variance explained (percent) is based on results reported for Bray–Curtis dissimilarity using multiple regression on distance matrices.

Weinstein et al. (3) examine the gut microbiomes of woodrats (*Neotoma* spp.), one of the best studied mammalian examples of a beneficial microbiota allowing host adaptation to novel diets (4). Woodrats are largely herbivorous, with varying degrees of specialization to certain plants that can be associated with microbiome variation. Different woodrat species often occur in the same habitats, such that environmental influences other than diet on the microbiome are minimized. In an unprecedented study design, Weinstein et al. combined field and laboratory experiments using 25 populations representing seven species. To fully characterize dietary variation, they used plant DNA metabarcoding and stable isotope analyses to quantify the diversity and relative abundances of plant species that were consumed by each individual woodrat. To standardize the effects of diet and environment, animals were taken into captivity for 1 mo on a common diet. Microbiomes of 123 individuals from all species were compared both in the wild and in captivity (Fig. 1).

In the wild, more genetically related individuals tend to harbor more similar microbial communities after accounting for geographic distance (3). Furthermore, a common diet was not enough to disturb the species-specific differences in the microbiota. Despite the significant shifts in microbiota from the wild to captivity, woodrat species retain the majority of their microbial taxa, as has also been observed in wild house mice (5, 6). This suggests that the mammalian host can maintain species- and population-specific microbiota through their host genetic factors and/or vertical transmission. Notably, changes in microbial richness before and after captivity significantly differ among the woodrat species. The species-specific responses of the microbiome to captivity further support the role of host genetics in microbiome structure.

Geography and neutral processes also explain some of the variation in the microbiota of wild woodrats (3). Taking advantage of species pairs that occur in the same and different locations, the study shows how microbiomes are more similar if the species live in the same place. The results suggest a microbial pool shared among different species that occur in the same habitat, but also highlights that some gut microbial taxa are geographically restricted (7). In addition, this is one of the few studies that tested for neutral processes (bacterial dispersal, stochastic processes, etc.). The authors categorized microbial taxa into “neutral” and “selected” based on deviations from the neutral distribution of microbial taxa (8, 9). A little over half of the taxa occurred at frequencies predicted by neutral assembly processes. Surprisingly, effects of geography were stronger for “neutral” microbes, and effects of phylogeny were stronger for “selected” microbes. The phylogenetic effect on microbes that deviate from the neutral distribution suggests that these “selected” microbes are dispersal limited or restricted to certain host species.

Gut microbes degrade plant polysaccharides, and the richness of fiber types within a diet predicts the richness of the gut microbiota (10, 11). This was also true for the woodrats (3). In this study, however, the authors characterize this relationship with unparalleled precision: One additional plant family consumed corresponds to approximately seven additional microbial taxa. The use of plant DNA metabarcoding in this study proves to be a powerful approach for characterizing dietary diversity, one that could be generally applied to the study of microbiomes of wild animals.

In PNAS, Weinstein et al. make a significant step toward untangling how host phylogeny, diet, and shared environment explain phylosymbiosis.

The authors (3) suggest that the relationship between dietary richness and microbial richness in woodrats could be maintained by selective pressures to digest diverse polysaccharides in the diet. The relationship could also be driven by transient microbes from diverse plant-associated microbial communities. Novel microbes or genes that are acquired through the environment may benefit host fitness, and beneficial microbes could then spread among hosts, allowing the population to adapt to a novel food source. For example, insects can acquire soil microbes that detoxify insecticides (12), and humans have acquired the ability to digest seaweeds through gut microbes that acquired genes from marine microbes (13). More empirical and theoretical studies are needed to better understand the mechanisms of microbiome-mediated host adaptation in complex systems such as the mammalian gut microbiota (14).

Overall, this innovative and comprehensive study supports the role of host genetics as a driver of phylosymbiosis in the wild and in captivity (3). Host genetic differences in physiology, immunity, gut morphology, and behavior can all result in host species-specific microbiota (15). Identifying the genetic basis of phylosymbiosis will provide mechanistic hypotheses on how mammalian hosts can recruit and exclude certain microbes to generate patterns of phylosymbiosis. Woodrats are a particularly powerful system because host genes and pathways that associate with species-specific features of the microbiota can be tested using gene editing in laboratory rodents. Furthermore, bacterial strain-level variation could be used to test whether host–microbial codiversification contributes to patterns of phylosymbiosis (16). Mechanisms underlying phylosymbiosis may not be mutually exclusive, and identifying host–microbial interactions that result in such patterns remains a challenge. This study by Weinstein et al. paves the way to a better understanding of ecological and evolutionary rules governing the relationship between hosts and their microbes.
